# Pneumococcal Carriage in Burkina Faso After 13-Valent Pneumococcal Conjugate Vaccine Introduction and Before a Schedule Change

**DOI:** 10.1093/ofid/ofae303

**Published:** 2024-05-31

**Authors:** Lana Childs, Issa Ouedraogo, Robert Lamoussa Zoma, T Félix Tarbangdo, Guetwendé Sawadogo, H Flavien Aké, Soumeya Ouangraoua, Soufiane Sanou, Theresa Tran, Srinivasan Velusamy, Tolulope Adebanjo, Chris A Van Beneden, Lesley McGee, Miwako Kobayashi

**Affiliations:** Infectious Disease Programs, CDC Foundation, Atlanta, Georgia, USA; Direction de la prévention par la vaccination, Ministère de la Santé et de l’Hygiène Publique, Ouagadougou, Burkina Faso; Davycas International, Ouagadougou, Burkina Faso; Davycas International, Ouagadougou, Burkina Faso; Davycas International, Ouagadougou, Burkina Faso; Davycas International, Ouagadougou, Burkina Faso; Unité de Bactériologie, Centre Muraz, Bobo-Dioulasso, Burkina Faso; Unité de Bactériologie, Centre Muraz, Bobo-Dioulasso, Burkina Faso; Division of Bacterial Diseases, National Center for Immunization and Respiratory Diseases, Centers for Disease Control and Prevention, Atlanta, Georgia, USA; Division of Bacterial Diseases, National Center for Immunization and Respiratory Diseases, Centers for Disease Control and Prevention, Atlanta, Georgia, USA; Division of Bacterial Diseases, National Center for Immunization and Respiratory Diseases, Centers for Disease Control and Prevention, Atlanta, Georgia, USA; Division of Bacterial Diseases, National Center for Immunization and Respiratory Diseases, Centers for Disease Control and Prevention, Atlanta, Georgia, USA; Division of Bacterial Diseases, National Center for Immunization and Respiratory Diseases, Centers for Disease Control and Prevention, Atlanta, Georgia, USA; Division of Bacterial Diseases, National Center for Immunization and Respiratory Diseases, Centers for Disease Control and Prevention, Atlanta, Georgia, USA

**Keywords:** Burkina Faso, carriage, 13-valent pneumococcal conjugate vaccine, pneumococcal vaccines, *Streptococcus pneumoniae*

## Abstract

**Background:**

In October 2013, Burkina Faso introduced 13-valent pneumococcal conjugate vaccine (PCV13) into the routine childhood immunization program using 3 primary doses with no booster. Previous pneumococcal carriage studies showed reductions in vaccine-type (VT) carriage in children aged <5 years but not in older age groups.

**Methods:**

We conducted a cross-sectional, age-stratified pneumococcal carriage study among healthy persons aged ≥1 month in Bobo-Dioulasso in March 2020. Pneumococci isolated by culture from nasopharyngeal swabs (all participants) and oropharyngeal swabs (participants aged ≥5 years) were serotyped by polymerase chain reaction; a subset was serotyped by Quellung. Using data from a study with the same design from March 2017, we examined changes in pneumococcal carriage by age group.

**Results:**

Among 1005 (2017) and 1002 (2020) enrolled participants, VT carriage decreased (21.6% to 15.9%; adjusted prevalence ratio [aPR], 0.76 [95% confidence interval {CI}, .63–.92]). By age group, decline in VT carriage was significant among children aged 5–14 years (28.9% to 16.3%; aPR, 0.57 [95% CI, .39–.84]) but not among children aged <5 years (22.4% to 19.1%; aPR, 0.87 [95% CI, .70–1.09]) or adults aged ≥15 years (12.0% to 5.5%; aPR, 0.52 [95% CI, .26–1.05]).

**Conclusions:**

Between 3 and 6 years after PCV13 introduction, significant declines in VT carriage were observed in older children, possibly reflecting indirect effects of PCV13 use. VT carriage in children aged <5 years remained stable with almost 1 in 5 carrying VT pneumococci, suggesting limitations to a PCV schedule without a booster dose.

## Teaser

We examined changes in the pneumococcal carriage prevalence among healthy persons aged ≥1 month in Burkina Faso between 3 and 6 years after 13-valent pneumococcal conjugate vaccine (PCV13) introduction and before a change in the PCV13 schedule.

*Streptococcus pneumoniae* (pneumococcus) is the most common bacterial cause of deaths among children aged <5 years globally [1]. In 2015, there were an estimated 9.2 million pneumococcal infections and 318 000 pneumococcus-related deaths among children aged <5 years worldwide; approximately half of the pneumococcus-related deaths occurred in Africa [2].

Widespread use of pneumococcal conjugate vaccines (PCVs) has led to decreased incidence in pneumococcal disease and declines in vaccine-type (VT) carriage among vaccinated children, resulting in decreased transmission of VTs from vaccinated children to unvaccinated children and adults (ie, herd protection) [2–6]. The World Health Organization (WHO) recommends the inclusion of PCVs in routine childhood immunization programs using a 3-dose schedule, administered as 3 primary doses without a booster (3 + 0) or 2 primary doses with a booster (2 + 1) depending on programmatic considerations of the country. The 2 + 1 schedule has potential benefits over the 3 + 0 schedule due to the booster dose inducing higher antibody levels in the second year of life, which may provide greater herd effects due to a longer duration of protection against carriage acquisition among vaccinated children [7].

Burkina Faso is a low-income country in sub-Saharan West Africa located entirely in the African meningitis belt and experiences hyperendemic rates of bacterial meningitis [8]. Following the introduction of *Haemophilus influenzae* serotype b vaccine in 2006 and meningococcal A (MenA) conjugate vaccine in 2010, *S pneumoniae* became the predominant cause of bacterial meningitis among all ages [8, 9]. Burkina Faso introduced 13-valent PCV (PCV13) into the childhood routine immunization program in October 2013 using a 3 + 0 schedule with doses administered at 2, 3, and 4 months of age with no catch-up campaigns. The WHO/United Nations Children's Fund national coverage estimate of the third dose of PCV13 has been >90% for most years following introduction [10].

Pre- (2008) and post-PCV13 introduction (2015, 2017) pneumococcal carriage studies conducted in Bobo-Dioulasso, Burkina Faso showed reductions in VTs among pneumococcal carriers aged <5 years, but not among participants ≥5 years [11]. Approximately 3 years post-PCV13 introduction (2017 study), VT carriage remained high among children aged <5 years (22%), older children aged 5–14 years (29%), and adults aged ≥15 years (12%) [11] compared to middle- and high-income countries using PCVs (children <5 years: 1.4%–11.2%; adults: 0.0%–9.0%) [12–15]. Nationwide bacterial meningitis surveillance showed declines in VT pneumococcal meningitis 4 years after vaccine introduction; however, serotype 1, included in PCV13, remained the predominant serotype causing pneumococcal meningitis among all ages [9]. In June 2021, Burkina Faso changed the PCV13 schedule to 2 + 1 with 2 primary doses administered at 2 and 4 months of age with a booster given at 9 months of age to improve protection against remaining vaccine-preventable pneumococcal disease.

We conducted a cross-sectional community pneumococcal carriage study among persons aged ≥1 month approximately 6 years post-PCV13 introduction to establish a baseline before the PCV13 schedule change and to examine changes in overall and VT pneumococcal carriage from the aforementioned 2017 study to 2020.

## METHODS

### Study Design and Participants

In March 2020, we conducted a cross-sectional, age-stratified community pneumococcal carriage study in Bobo-Dioulasso, a city located in Western Burkina Faso. We used the same recruiting methods as previous pneumococcal carriage studies conducted in Bobo-Dioulasso pre- and post-PCV13 introduction (2008, 2015, 2017) [11, 16] and compared with data from the 2017 survey. Target sample size for each study (2017 and 2020) was 1000 persons aged ≥1 month with 200 participants each in 5 age groups: 1–11 months, 1 year, 2–4 years, 5–14 years, and ≥15 years.

We randomly selected 10 sectors among 21 eligible sectors (military and industrial sectors excluded) with 2 back-up sectors; in each sector, 20 crossroads were randomly selected with 10 back-up crossroads. At each crossroad, a street was randomly selected, and households were visited starting with the first on the left. The aim was to recruit 1 person in each age group at each crossroad to reach the target sample size. If there was >1 person in the same age group living in the same household, 1 was randomly selected. People living in the same household, but of separate age groups, were eligible for inclusion. The inclusion criteria for participants were resident of Bobo-Dioulasso, aged ≥1 month, and informed consent of an adult participant or of the parent/guardian of a child aged <18 years. The exclusion criteria were residence outside of Bobo-Dioulasso, severe malnutrition, or severe underlying disease.

### Data and Specimen Collection

Data collection methods have been previously described [11]. In brief, surveyors collected information on household characteristics and demographics from all consenting participants. Vaccination history was collected from children aged <5 years in 2017 and <7 years in 2020 using vaccination cards. The study team reviewed immunization registers in local health posts to collect vaccine histories for children without vaccination cards during enrollment. Enrolled participants were given appointment cards to visit a district hospital for clinical specimen collection.

At the district hospital, informed consent for clinical specimen collection was obtained and a second questionnaire was administered that included questions on the health history of participants. Trained nurses collected nasopharyngeal swabs from all participants and oropharyngeal swabs from participants aged ≥5 years [17]. Swabs were immediately placed into cryotubes containing 1 mL skim milk, tryptone, glucose, and glycerol (STGG) transport medium and placed in coolers with ice packs. Specimens were transported to Centre Muraz, a national reference laboratory in Bobo-Dioulasso, within 4–6 hours.

### Laboratory Methods

When specimens arrived at Centre Muraz, inoculated STGG was vortexed for 10–20 seconds before being stored in a −80°C freezer. For nasopharyngeal and oropharyngeal swab analysis, 200 µL of swab-inoculated STGG media was transferred to 5.0 mL Todd Hewitt broth containing 0.5% yeast extract (THY) and 1 mL of rabbit serum and incubated at 35°C–37°C for 6 hours. Cultured broth was plated on sheep blood agar and incubated in 5% carbon dioxide at 35°C–37°C. After 18–24 hours of incubation, plates were examined for the appearance of α-hemolytic colonies resembling streptococci. Pneumococci were identified by susceptibility to optochin and bile solubility test. *S pneumoniae* isolates were inoculated in a preservation medium STGG and stored at −80°C.

Pneumococcal serotypes were determined using published sequential multiplex polymerase chain reaction (PCR) assay [18]. Quality control of the bacterial identification results obtained by Centre Muraz was performed at the Centers for Disease Control and Prevention (CDC) *Streptococcus* Laboratory in Atlanta, Georgia, for 20% of negative samples in 2017 and all negative samples in 2020. Pneumococcal isolates determined to be nontypeable (NT) or for which the serotype was unresolved by multiplex PCR were tested by Quellung reaction at CDC. Additionally, 20% of serotyped isolates were sent to CDC for quality control of the results obtained by Centre Muraz.

### Data Analysis

#### Definitions

PCV13 serotypes were considered VTs: 1, 3, 4, 5, 6A, 6B, 7F, 9V, 14, 18C, 19A, 19F, and 23F. All other serotypes were considered nonvaccine types (NVTs), excluding nontypeables.

PCV13 doses were considered valid if children were aged ≥2 months for dose 1, each dose was given ≥21 days apart, and ≥14 days occurred between a dose and clinical specimen collection.

#### Data analyses

We conducted descriptive analyses of participants by study year and age group. We used χ^2^ tests and Fisher exact tests to compare proportions. Changes in the overall, VT, and NVT carriage prevalence were examined between 2017 and 2020 among all participants and by age group. The carriage prevalence of individual serotypes in 2017 was compared to prevalence in 2020 for all age groups and all children aged <5 years combined; individuals carrying >1 serotype were counted in the numerator for each serotype. Crude prevalence ratios (PRs) were calculated using standard methods with 2017 serving as the reference period. We reviewed literature to identify potential confounders associated with pneumococcal carriage (inside cooking location, presence of other children aged <5 years in the household, antibiotic use in the past 2 weeks, and illness in the past 2 weeks) for a priori inclusion in the adjusted model [11, 19–22]. We also looked at participant characteristics that changed from 2017 and 2020 (Table 1) and whether there were any significant differences in pneumococcal carriage prevalence by those characteristics (Supplementary Table 1) to inform our model selection. Adjusted prevalence ratios (aPRs) were modeled using log-binomial regression. Poisson regression using robust error variance was used if log-binomial models failed to converge [23, 24]. Data analyses were performed using SAS software (version 9.4). *P* values <.05 were considered statistically significant.

**Table 1.** Demographic and Epidemiological Characteristics of Enrolled Participants by Study Year—Bobo-Dioulasso, Burkina Faso, 2017 and 2020

Characteristic	2017 (N = 1005)		2020 (N = 1002)		*P* Value^a^
	No.	(%)	No.	(%)	
Age, y					
<1	201	(20.0)	200	(20.0)	.999
1	199	(19.8)	200	(20.0)	
2–4	204	(20.3)	201	(20.1)	
5–14	201	(20.0)	202	(20.2)	
≥15	200	(19.9)	199	(19.9)	
Female sex	559	(55.6)	548	(54.7)	.675
Presence of other children <5 y of age in the household (other than the participant)^b^	943	(93.9)	889	(88.7)	<.0001
Household size^b^					
1–3	149	(14.8)	141	(14.1)	<.0001
4–6	423	(42.1)	519	(51.8)	
>6	432	(43.0)	342	(34.1)	
≥4 persons sharing a room^c^	151	(15.1)	133	(13.3)	.249
Children in household attending daycare or school^c^	763	(76.1)	777	(77.5)	.458
Cigarette smoker in household^c^	208	(20.8)	152	(15.2)	.001
Fuel source^d^					
Gas	312	(31.0)	115	(11.5)	
Coal	835	(83.1)	709	(70.8)	
Wood	488	(48.6)	178	(17.8)	
Cooking location					
Inside	304	(30.2)	455	(45.4)	<.0001
Under hangar	141	(14.0)	141	(14.1)	.978
Outside	757	(75.3)	662	(66.1)	<.0001
Household possessions					
Radio	753	(74.9)	717	(71.6)	.088
Television	830	(82.6)	885	(88.3)	.0003
Phone	983	(97.8)	996	(99.4)	.002
Motorbike	810	(80.6)	863	(86.1)	.0009
Illness in the past 2 wk					
Cold/runny nose	474	(47.2)	531	(53.0)	.009
Cough	376	(37.4)	297	(29.6)	.0002
Fever	208	(20.7)	143	(14.3)	.0002
Antibiotic use in the past 2 wk	114	(11.3)	129	(12.9)	.293

^a^χ^2^ tests were used to compare 2017 versus 2020 categorical responses.

^b^One response missing from 2017 study (N = 1004).

^c^Three responses missing from 2017 study (N = 1002).

^d^Multiple responses possible in 2017 study while 1 response only possible in 2020 study. *P* value not calculated due to differences in the question format.

### Patient Consent Statement

Prior to enrollment at the household, the study objectives were explained to participants in French or the local language, and written consent was obtained from all participants. At the district hospital, informed consent was obtained prior to the collection of clinical specimens. The study documents were approved by the Burkina Faso ethical committee. This activity was reviewed by CDC, deemed not research, and was conducted consistent with applicable federal law and CDC policy (see, eg, 45 Code of Federal Regulations [C.F.R.] part 46.102(I)(2), 21 C.F.R. part 56: 42 United States Code [U.S.C.] §241(d); 5 U.S.C. §552a; 44 U.S.C. §3501 et seq). Data collection for the study conducted in March 2020 was completed prior to the implementation of coronavirus disease 2019 risk mitigations in Burkina Faso.

## RESULTS

### Household and Participant Characteristics

We enrolled a total of 2007 participants (2017: n = 1005; 2020: n = 1002) in the 2 studies (Table 1). Compared to 2017, there were fewer households in 2020 with presence of other children aged <5 years (88.7% vs 93.9%; *P* < .0001), >6 household members (34.1% vs 43.0%; *P* < .0001), and a cigarette smoker (15.2% vs 20.8%; *P* = .001). More households in 2020 cooked inside (45.4% vs 30.2%; *P* < .0001) and had a television (88.3% vs 82.6%; *P* = .0003), phone (99.4% vs 97.8%; *P* = .002), or motorbike (86.1% vs 80.6%; *P* = .0009) than in 2017. Participants in 2017 compared to 2020 were more likely to have a cough (37.4% vs 29.6%; *P* = .0002) or fever (20.7% vs 14.3%; *P* = .0002) and less likely to have a cold/runny nose (47.2% vs 53.0%; *P* = .009) in the past 2 weeks.

### Overall Pneumococcal Carriage by Household and Participant Characteristics

In 2017 and 2020, households with presence of other children aged <5 years or with ≥4 persons sharing a room and participants with a cold/runny nose or cough had a significantly higher pneumococcal carriage prevalence than those without (Supplementary Table 1). Households using gas as a fuel source had a significantly lower pneumococcal carriage prevalence than households not using gas in both study years. There were no significant changes in the overall pneumococcal carriage prevalence by household and participant characteristics from 2017 to 2020.

### Overall Pneumococcal Carriage Prevalence

Among participants of all ages, there were no significant changes in overall pneumococcal carriage prevalence from 2017 to 2020 (60.6% to 59.3%; aPR, 1.02 [95% confidence interval {CI}, .95–1.09]) (Table 2). By age group, pneumococcal carriage prevalence declined among children aged 5–14 years (65.2% to 53.0%; aPR, 0.83 [95% CI, .70–.98]) and adults (37.0% to 25.1%; aPR, 0.75 [95% CI, .56–1.02]), although the changes in adults were not significant. Pneumococcal carriage prevalence was highest among children aged 1 year in both 2017 (73.4%) and 2020 (75.5%).

**Table 2.** Pneumococcal Carriage Prevalence and Prevalence Ratios by Age Group—Bobo-Dioulasso, Burkina Faso, 2017 and 2020

Characteristic	2017 Carriage Prevalence		2020 Carriage Prevalence		Crude PR^a^	Adjusted PR^b^
	no./No.	% (95% CI)	no./No.	% (95% CI)	(95% CI)	(95% CI)
Overall pneumococcal carriage						
<1 y	129/201	64.2 (57.5–70.8)	139/200	69.5 (63.1–75.9)	1.08 (.94–1.24)	1.08 (.94–1.23)
1 y	146/199	73.4 (67.2–79.5)	151/200	75.5 (69.5–81.5)	1.03 (.92–1.15)	1.05 (.94–1.18)
2–4 y	129/204	63.2 (56.6–69.9)	147/201	73.1 (67.0–79.3)	1.16 (1.01–1.32)	1.15 (1.00–1.31)
5–14 y	131/201	65.2 (58.6–71.8)	107/202	53.0 (46.1–59.9)	0.81 (.69–.96)	0.83 (.70–.98)
≥15 y	74/200	37.0 (30.3–43.7)	50/199	25.1 (19.1–31.2)	0.68 (.50–.92)	0.75 (.56–1.02)
All ages	609/1005	60.6 (57.6–63.6)	594/1002	59.3 (56.2–62.3)	0.98 (.91–1.05)	1.02 (.95–1.09)
VT carriage^c^ among all participants						
<1 y	48/201	23.9 (18.0–29.8)	38/200	19.0 (13.6–24.4)	0.80 (.55–1.16)	0.80 (.55–1.16)
1 y	41/199	20.6 (15.0–26.2)	39/200	19.5 (14.0–25.0)	0.95 (.64–1.40)	1.02 (.69–1.52)
2–4 y	46/204	22.5 (16.8–28.3)	38/201	18.9 (13.5–24.3)	0.84 (.57–1.23)	0.85 (.57–1.25)
5–14 y	58/201	28.9 (22.6–35.1)	33/202	16.3 (11.2–21.4)	0.57 (.39–.83)	0.57 (.39–.84)
≥15 y	24/200	12.0 (7.5–16.5)	11/199	5.5 (2.3–8.7)	0.46 (.23–.91)	0.52 (.26–1.05)
All ages	217/1005	21.6 (19.0–24.1)	159/1002	15.9 (13.6–18.1)	0.73 (.61–.88)	0.76 (.63–.92)
VT carriage among pneumococcal carriers^c^						
<1 y	48/129	37.2 (28.8–45.6)	38/139	27.3 (19.9–34.8)	0.73 (.52–1.04)	0.70 (.49–.98)
1 y	41/146	28.1 (20.8–35.4)	39/151	25.8 (18.8–32.8)	0.92 (.63–1.34)	0.96 (.66–1.40)
2–4 y	46/129	35.7 (27.4–43.9)	38/147	25.9 (18.8–32.9)	0.72 (.51–1.04)	0.73 (.47–1.12)
5–14 y	58/131	44.3 (35.7–52.8)	33/107	30.8 (22.1–39.6)	0.70 (.49–.98)	0.75 (.50–1.00)
≥15 y	24/74	32.4 (21.7–43.1)	11/50	22.0 (10.5–33.5)	0.68 (.37–1.26)	0.67 (.36–1.30)
All ages	217/609	35.6 (31.8–39.4)	159/594	26.8 (23.2–30.3)	0.75 (.63–.89)	0.75 (.64–.90)
NVT carriage^d^ among all participants						
<1 y	78/201	38.8 (32.1–45.6)	90/200	45.0 (38.1–51.9)	1.16 (.92–1.46)	1.12 (.89–1.40)
1 y	96/199	48.2 (41.3–55.2)	104/200	52.0 (45.1–58.9)	1.08 (.89–1.31)	1.09 (.89–1.33)
2–4 y	70/204	34.3 (27.8–40.8)	97/201	48.3 (41.3–55.2)	1.41 (1.11–1.78)	1.41 (1.11–1.79)
5–14 y	71/201	35.3 (28.7–41.9)	70/202	34.7 (28.1–41.2)	0.98 (.75–1.28)	0.98 (.75–1.28)
≥15 y	46/200	23.0 (17.2–28.8)	36/199	18.1 (12.7–23.4)	0.79 (.53–1.16)	0.84 (.57–1.26)
All ages	361/1005	35.9 (32.9–38.9)	397/1002	39.6 (36.6–42.7)	1.10 (.99–1.23)	1.13 (1.01–1.27)

Abbreviations: CI, confidence interval; NT, nontypeable; NVT, nonvaccine type; PR, prevalence ratio; VT, vaccine type.

^a^Standard methods for calculating risk ratios were used to obtain crude PRs, with 2017 serving as the reference period.

^b^Adjusted PRs were modeled using log-binomial regression. Poisson regression using robust error variance was used if log-binomial models failed to converge [23, 24]. Models were adjusted for inside cooking location, presence of other children aged <5 years in the household, antibiotic use in the past 2 weeks, and illness in the past 2 weeks (cough, cold/runny nose, fever).

^c^VT carriage is defined as carriage with serotypes included in 13-valent pneumococcal conjugate vaccine (PCV13) (1, 3, 4, 5, 6A, 6B, 7F, 9V, 14, 18C, 19A, and 23F).

^d^NVT carriage is defined as carriage with any serotype not included in PCV13, excluding NTs.

### VT Pneumococcal Carriage Prevalence

Among all pneumococcal carriers, VT carriage declined significantly (35.6% to 26.8%; aPR, 0.75 [95% CI, .64–.90]), although by age group, the changes were only significant in children aged <1 year (37.2% to 27.3%; aPR, 0.70 [95% CI, .49–.98]) (Table 2). Among all participants, VT carriage decreased from 21.6% in 2017 to 15.9% in 2020 (aPR, 0.76 [95% CI, .63–.92]) (Table 2). VT carriage was the highest among children aged 5–14 years (28.9%) in 2017 and children aged 1 year (19.5%) in 2020. By age group, the changes in VT carriage between 2017 and 2020 were only significant in children aged 5–14 years (28.9% to 16.3%; aPR, 0.57 [95% CI, .39–.84]). Of note, VT carriage declined among adults from 12.0% in 2017 to 5.5% in 2020, though the changes were not significant (aPR, 0.52 [95% CI, .26–1.05]).

### Serotype Distribution

**Figure 1.**
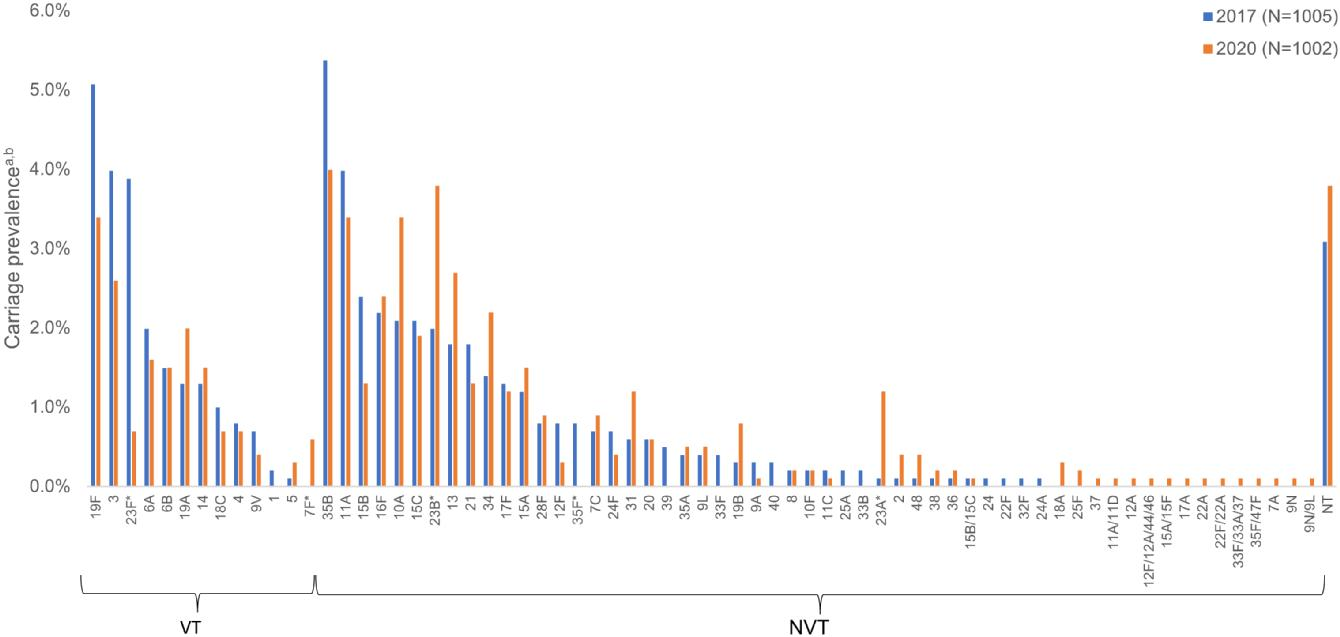
Pneumococcal carriage prevalence by serotype among all ages in 2017 (N = 1005) and 2020 (N = 1002), Bobo-Dioulasso, Burkina Faso. *P < .05. ^a^χ^2^ tests and Fisher exact tests were used to compare changes in individual vaccine serotype carriage prevalence in each age group between 2017 and 2020. ^b^Sixteen participants in 2017 and 5 participants in 2020 were colonized with >1 serotype.

**Figure 2.**
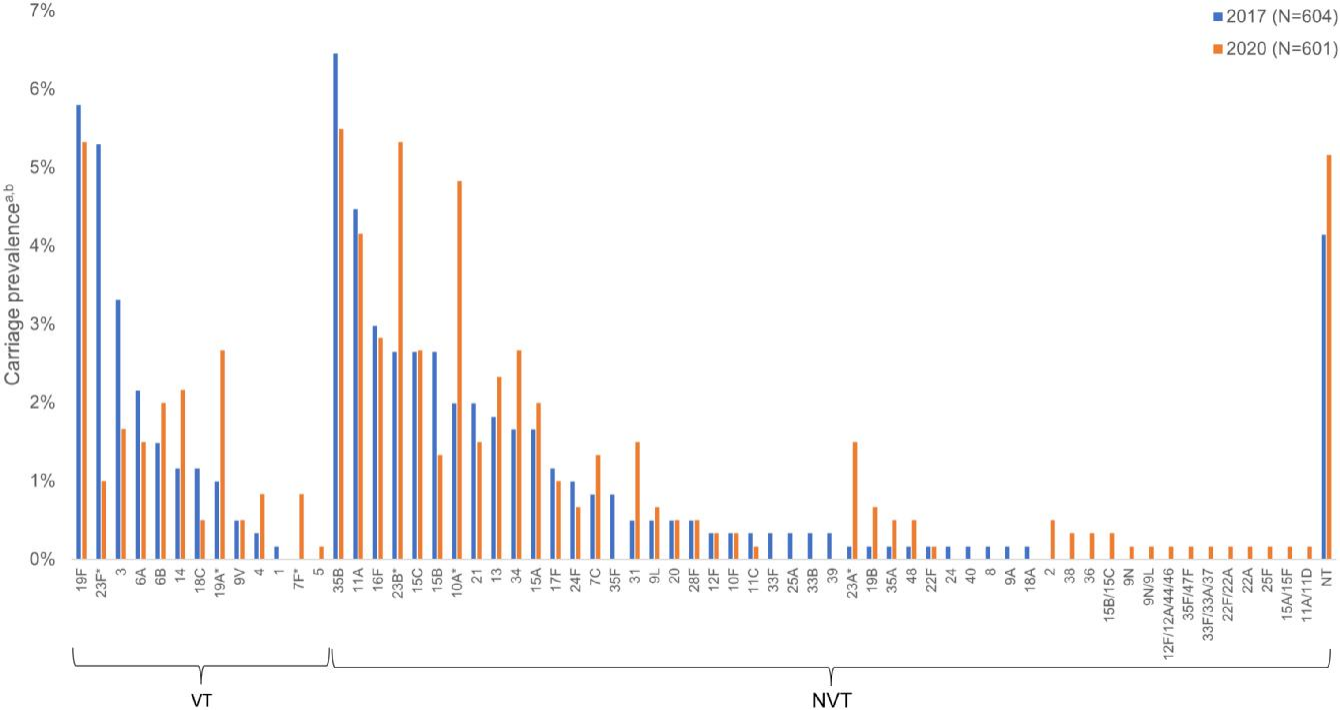
Pneumococcal carriage prevalence by serotype among children aged <5 y in 2017 (N = 604) and 2020 (N = 601), Bobo-Dioulasso, Burkina Faso. *P < .05. ^a^χ^2^ tests and Fisher exact tests were used to compare changes in individual vaccine serotype carriage prevalence in each age group between 2017 and 2020. ^b^Sixteen participants in 2017 and 5 participants in 2020 were colonized with >1 serotype. Abbreviations: NT, nontypeable; NVT, nonvaccine type; VT, vaccine type.

In 2020, the VTs with the highest carriage prevalence in participants of all ages were serotypes 19F (3.4%), 3 (2.6%), and 19A (2.0%) (Figure 1), with serotypes 19A and 19F being the most common among children aged <5 years (Figure 2) and serotype 3 being the most common among participants aged ≥5 years (Supplementary Table 2). The NVTs with the highest carriage prevalence in participants of all ages were serotypes 35B (4.0%), 23B (3.8%), and 11A (3.4%) (Figure 1).

Between 2017 and 2020, carriage prevalence in participants of all ages decreased significantly for serotype 23F (VT) (2017: 3.9%; 2020: 0.7%) and serotype 35F (NVT) (2017: 0.8%; 2020: 0.0%), and increased significantly for serotypes 7F (VT) (2017: 0.0%; 2020: 0.6%), 23B (NVT) (2017: 2.0%; 2020: 3.8%), and 23A (NVT) (2017: 0.1%; 2020: 1.2%) (Figure 1). From 2017 to 2020, carriage prevalence in children aged <5 years decreased significantly for serotype 23F (2017: 5.3%; 2020: 1.0%), and serotypes 19A (2017: 1.0%; 2020: 2.7%) and 7F (2017: 0.0%; 2020: 0.8%) increased significantly (Figure 2). Carriage prevalence of serotype 19F did not change significantly in children aged <5 years, whereas significant decreases were observed in children aged 5–14 years (Supplementary Table 2). Among children aged <5 years, the prevalence of NVTs 23B, 10A, and 23A increased significantly (Figure 2).

### PCV13 Vaccination History

Card- or registry-confirmed PCV13 vaccination history was available for 64.3% (387/602) of children aged <5 years in 2017 and 80.7% (536/664) of children aged <7 years in 2020 (Supplementary Table 3). Among children aged <5 years with confirmed vaccination history, the proportion who received 3 valid PCV13 doses increased from 64.3% in 2017 to 79.2% in 2020 (Table 3). By age group, 3-dose PCV13 coverage was 50.3% and 60.1% among children aged 2–11 months (not all children in this age group would have been age eligible to complete the PCV13 series), 84.5% and 92.4% among children aged 1 year, and 60.4% and 84.6% among children aged 2–4 years in 2017 and 2020, respectively. In 2020, 71.7% of children aged 5–6 years had received 3 valid PCV13 doses, representing 16.3% (33/202) of children enrolled in the age group 5–14 years.

**Table 3.** Number of Valid 13-Valent Pneumococcal Conjugate Vaccine Doses by Age Group Among Children Aged <5 Years in 2017 and <7 Years in 2020—Bobo-Dioulasso, Burkina Faso, 2017 and 2020

No. of Valid PCV13 Doses^a^	Age Group^b^ and Study Year							
	2–11 mo		1 y		2–4 y		5–6 y	
	2017 (n = 189)	2020 (n = 178)	2017 (n = 162)	2020 (n = 185)	2017 (n = 139)	2020 (n = 175)	2017^c^	2020^d^ (n = 46)
0	47 (24.9)	23 (12.9)	16 (9.9)	7 (3.8)	45 (32.4)	19 (10.9)	…	10 (21.7)
1	27 (14.3)	20 (11.2)	3 (1.9)	0 (0.0)	2 (1.2)	3 (1.7)	…	0 (0.0)
2	20 (10.6)	28 (15.7)	7 (4.3)	7 (3.8)	8 (5.8)	5 (2.9)	…	3 (6.5)
3	95 (50.3)	107 (60.1)	136 (84.0)	171 (92.4)	84 (60.4)	148 (84.6)	…	33 (71.7)

Data are presented as No. (%).

Abbreviation: PCV13, 13-valent pneumococcal conjugate vaccine.

^a^PCV13 doses were considered valid if children were ≥2 months for PCV13 dose 1, each dose was given ≥21 days apart, and ≥14 days occurred between a PCV13 dose and collection of the nasopharyngeal and oropharyngeal (participants ≥5 years) swab.

^b^The vaccination status of some participants was unknown, did not meet the criteria to be considered valid, or was given only by caregiver verbal report, so the total number of children under each column does not necessarily add up to the number of enrolled children in each age group.

^c^Since PCV13 was introduced in 2013, children ≥5 years were not eligible for PCV13 vaccination in 2017.

^d^Children aged 5 and 6 years are included in the age group 5–14 years. There were 65 children 5 or 6 years old enrolled in 2020.

## DISCUSSION

We examined changes between 2 pneumococcal carriage studies conducted approximately 3 and 6 years after PCV13 introduction in Burkina Faso and before a change in vaccine schedule. We found no significant change in overall pneumococcal carriage (60.6% to 59.3%) whereas VT carriage decreased significantly (21.6% to 15.9%) in participants of all ages, although by age group, declines were only significant among children aged 5–14 years. Despite increased PCV13 coverage over time, in 2020, VT carriage was observed in 1 in 5 children aged <5 years and remains high in all age groups compared to middle- and high-income countries using schedules with a booster dose [14, 25].

A previous report comparing carriage studies conducted in Bobo-Dioulasso in 2015 (1 year post–PCV13 introduction) and 2017 (3 years post-PCV13 introduction, also included in the current study) showed reductions in VT carriage among pneumococcal carriers aged <5 years, although there were differences in the laboratory methods used for pneumococcal detection between the studies conducted in 2015 and 2017 [11]. Between 2017 and 2020, we observed no further significant reduction in VT carriage among children aged <5 years. Persistent VT carriage has been observed in other sub-Saharan countries using the 3 + 0 schedule. In the Gambia, 5 years post–PCV13 introduction (preceded by 7-valent PCV introduction 2 years prior), VT carriage was 13.5%–14.4% in children aged <5 years [26]; in Malawi, 7 years post–PCV13 introduction, VT carriage was 15.7%–16.7% among children aged 3–8 years [22]. In Mozambique, VT carriage was 14.5% in human immunodeficiency virus (HIV)–uninfected children and 21.0% in HIV-infected children 3 years after 10-valent PCV introduction [27]. Persistent VT carriage in younger children results in less pronounced indirect effects on carriage and disease for older ages. Preliminary meningitis surveillance data from Burkina Faso from 2018 through 2020 showed no significant declines in VT pneumococcal meningitis incidence for all age groups combined, and serotype 1, a PCV13 serotype rarely detected in carriage, remained the predominant serotype causing pneumococcal meningitis among older children and adults [28]. High residual VT carriage and disease found in sub-Saharan countries, including Burkina Faso, prompted evaluations of strategies to optimize PCV impact including alternative schedules such as those with a booster. In 2019, WHO issued a new position paper stating that the 2 + 1 schedule could potentially provide a longer duration of protection than 3 + 0, although data are still limited [7].

The aforementioned report comparing results from early post–PCV13 introduction (2015 vs 2017) in Bobo-Dioulasso found no clear evidence of reductions in VT carriage in older children or adults [11]. The reductions in VT carriage we observed in children aged 5–14 years (some of whom were PCV13 vaccinated in 2020) and adults (nonsignificant after adjustment) align with previous studies suggesting that it may take several years to develop herd protection. In Malawi, reductions in VT carriage among PCV13-unvaccinated HIV-infected adults were seen at 7 years post–PCV13 introduction using a 3 + 0 schedule (VT carriage 15.2% and 8.9%, 3.6 and 7.1 years post–PCV13 introduction, respectively) [22]. Additionally, a systematic review of studies in high- and middle-income countries found that the mean time to reach a 50% and 90% reduction in invasive pneumococcal disease (IPD) among unvaccinated populations was approximately 2–4 years and 9–10 years (depending on PCV product) post-PCV introduction, respectively [6]. Thus, the observed VT carriage reductions in persons aged ≥5 years could be explained by additional years of sustained PCV13 use in the community, with additional birth cohorts receiving PCV13.

In 2020, serotypes 3, 19A, and 19F were the most common VTs identified, with significant increases in the carriage prevalence of serotype 19A from 2017 (1.0%) to 2020 (2.7%) in children aged <5 years. In South Africa, serotypes 19A and 3 were the most identified VTs in IPD patients of all ages in the vaccine era, though neither serotype was significantly associated with increased in-hospital death [29]. Serotype 19A has not been a major cause of pneumococcal meningitis in Burkina Faso [9], but additional monitoring for carriage and disease is warranted, especially after the PCV13 schedule change.

As expected, we observed increases in NVT carriage from 2017 to 2020 in children <5 years of age. Serotype replacement following PCV introduction has been well documented [26, 30–32]. Specifically, we observed significant increases in serotypes 23B and 23A carriage among children aged <5 years and participants of all ages and serotype 10A among children aged <5 years only. Serotype 35B also remained as the most common NVT in participants of all ages and children aged <5 years in 2020. From 2011 to 2017, few pneumococcal meningitis cases caused by these NVTs have been reported [9]. A systematic review estimating the IPD potential of individual serotypes in children aged <5 years in the PCV era found that serotypes 23B and 35B had a lower IPD potential when serotype 19A was used as the reference [4]; however, additional monitoring of serotype 23B and 35B carriage and disease is needed since some countries have reported increases in IPD due to these serotypes [33, 34].

Our study is subject to several limitations. First, we did not account for the study cluster design or include sampling weights because we did not use a true probabilistic sampling design [35]. While sectors and crossroads were randomly selected, back-up sectors and crossroads had a chance of being selected twice. We compared the carriage prevalence in sectors selected during the first round with the back-up sectors and found no differences in the carriage prevalence when stratified by age group. Additionally, at each crossroad the first household on the left was visited and surveyors moved consecutively to subsequent households, possibly leading to a bias if participants living closest to crossroads were more or less likely to be pneumococcal carriers. Second, fewer adult males (2017: 19.5%; 2020: 35.2%) were enrolled; however, a systematic review found no association between participant sex and pneumococcal carriage [19]. Third, there could be residual unmeasured confounders contributing to significant reductions in overall pneumococcal carriage in children aged 5–14 years, therefore leading to significant reductions in VT carriage in this age group. Last, since we applied the same sample size for each age group, we may have been underpowered to detect significant changes in VT carriage in adults.

We observed sustained direct effects and the first evidence of indirect effects of PCV13 introduction on VT carriage in Burkina Faso. Despite declines in VT carriage from 2017 to 2020 among participants of all ages, there were no further significant reductions in children aged <5 years observed early post–PCV13 introduction [11], and VT carriage remained high. Approximately 6 years post-PCV13 introduction and in the context of high PCV13 coverage, 1 in 5 children aged <5 years still carries a VT pneumococci. Our study provides baseline VT carriage estimates before the PCV13 schedule change that occurred in June 2021. Additional monitoring of VT carriage is needed in the years following the schedule change to assess the impact. Evidence generated from future studies in Burkina Faso may inform pneumococcal vaccination policy decision-making for other countries in Africa using the 3 + 0 schedule.

## Supplementary Data

Supplementary materials are available at *Open Forum Infectious Diseases* online. Consisting of data provided by the authors to benefit the reader, the posted materials are not copyedited and are the sole responsibility of the authors, so questions or comments should be addressed to the corresponding author.

## Supplementary Material

ofae303_Supplementary_Data

## Notes

***Acknowledgments.*** The authors thank the study participants, their parents/guardians, the community of Bobo-Dioulasso, the Do and Dafra district hospitals, and the field staff for their participation in the studies. The authors also acknowledge Fernanda Lessa and Tamara Pilishvili for their support and input.

***Author contributions.*** L. C., I. O., R. L. Z., H. F. A., C. V. B., L. M., and M. K. conceptualized and designed the study. R. L. Z., T. F. T., G. S., H. F. A., and S. O. supervised data collection. T. A. and L. C. cleaned the 2017 and 2020 data, respectively. S. O., S. S., and T. T. conducted laboratory analyses. S. V. and L. M. reviewed and validated the laboratory analysis. L. C. did the statistical analysis and drafted the manuscript. All authors assisted with critical review of the manuscript. M. K. and C. V. B. provided expert opinion on study design, reviewed the manuscript, and critically analyzed the results.

***Financial support.*** The 2020 study was supported through a grant from the Bill & Melinda Gates Foundation (OPP1209963).

## References

[1] GBD 2019 Antimicrobial Resistance Collaborators . Global mortality associated with 33 bacterial pathogens in 2019: a systematic analysis for the Global Burden of Disease Study 2019. Lancet 2022; 400:2221–48. 36423648 10.1016/S0140-6736(22)02185-7 PMC9763654

[2] Wahl B , O’Brien KL, Greenbaum A, et al Burden of *Streptococcus pneumoniae* and *Haemophilus influenzae* type b disease in children in the era of conjugate vaccines: global, regional, and national estimates for 2000–15. Lancet Glob Health 2018; 6:e744–57. 29903376 10.1016/S2214-109X(18)30247-X PMC6005122

[3] Conklin L , Loo JD, Kirk J, et al Systematic review of the effect of pneumococcal conjugate vaccine dosing schedules on vaccine-type invasive pneumococcal disease among young children. Pediatr Infect Dis J 2014; 33(Suppl 2):S109–18. 24336053 10.1097/INF.0000000000000078 PMC3944481

[4] Balsells E , Dagan R, Yildirim I, et al The relative invasive disease potential of *Streptococcus pneumoniae* among children after PCV introduction: a systematic review and meta-analysis. J Infect 2018; 77:368–78. 29964140 10.1016/j.jinf.2018.06.004

[5] Hammitt LL , Bruden DL, Butler JC, et al Indirect effect of conjugate vaccine on adult carriage of *Streptococcus pneumoniae*: an explanation of trends in invasive pneumococcal disease. J Infect Dis 2006; 193:1487–94. 16652275 10.1086/503805

[6] Shiri T , Datta S, Madan J, et al Indirect effects of childhood pneumococcal conjugate vaccination on invasive pneumococcal disease: a systematic review and meta-analysis. Lancet Glob Health 2017; 5:e51–9. 27955789 10.1016/S2214-109X(16)30306-0

[7] World Health Organization . Pneumococcal conjugate vaccines in infants and children under 5 years of age: WHO position paper. Wkly Epidemiol Rec 2019; 94:85–104.

[8] Soeters HM , Diallo AO, Bicaba BW, et al Bacterial meningitis epidemiology in five countries in the meningitis belt of sub-Saharan Africa, 2015–2017. J Infect Dis 2019; 220(Suppl 4):S165–74. 31671441 10.1093/infdis/jiz358 PMC6853282

[9] Soeters HM , Kambire D, Sawadogo G, et al Impact of 13-valent pneumococcal conjugate vaccine on pneumococcal meningitis, Burkina Faso, 2016–2017. J Infect Dis 2019; 220(Suppl 4):S253–62. 31671444 10.1093/infdis/jiz301 PMC8935360

[10] World Health Organization . WHO/UNICEF estimates of national immunization coverage 2023. Available at: https://www.who.int/teams/immunization-vaccines-and-biologicals/immunization-analysis-and-insights/global-monitoring/immunization-coverage/who-unicef-estimates-of-national-immunization-coverage. Accessed 10 August 2023.

[11] Kabore L , Adebanjo T, Njanpop-Lafourcade BM, et al Pneumococcal carriage in Burkina Faso after 13-valent pneumococcal conjugate vaccine introduction: results from 2 cross-sectional population-based surveys. J Infect Dis 2021; 224(12 Suppl 2):S258–66. 34469552 10.1093/infdis/jiab037 PMC8409529

[12] Fleming-Dutra KE , Conklin L, Loo JD, et al Systematic review of the effect of pneumococcal conjugate vaccine dosing schedules on vaccine-type nasopharyngeal carriage. Pediatr Infect Dis J 2014; 33(Suppl 2):S152–60. 24336057 10.1097/INF.0000000000000083 PMC3940522

[13] Bosch A , van Houten MA, Bruin JP, et al Nasopharyngeal carriage of *Streptococcus pneumoniae* and other bacteria in the 7th year after implementation of the pneumococcal conjugate vaccine in the Netherlands. Vaccine 2016; 34:531–9. 26667610 10.1016/j.vaccine.2015.11.060

[14] Flasche S , Van Hoek AJ, Sheasby E, et al Effect of pneumococcal conjugate vaccination on serotype-specific carriage and invasive disease in England: a cross-sectional study. PLoS Med 2011; 8:e1001017. 21483718 10.1371/journal.pmed.1001017 PMC3071372

[15] Tiley KS , Ratcliffe H, Voysey M, et al Nasopharyngeal carriage of pneumococcus in children in England up to 10 years after 13-valent pneumococcal conjugate vaccine introduction: persistence of serotypes 3 and 19A and emergence of 7C. J Infect Dis 2023; 227:610–21. 36130327 10.1093/infdis/jiac376 PMC9978316

[16] Mueller JE , Yaro S, Ouedraogo MS, et al Pneumococci in the African meningitis belt: meningitis incidence and carriage prevalence in children and adults. PLoS One 2012; 7:e52464. 23285051 10.1371/journal.pone.0052464 PMC3527509

[17] Satzke C , Turner P, Virolainen-Julkunen A, et al Standard method for detecting upper respiratory carriage of *Streptococcus pneumoniae*: updated recommendations from the World Health Organization pneumococcal carriage working group. Vaccine 2013; 32:165–79. 24331112 10.1016/j.vaccine.2013.08.062

[18] Centers for Disease Control and Prevention *Streptococcus* Laboratory. *Streptococcus pneumoniae*: resources and protocols. 2021. Available at: https://www.cdc.gov/streplab/pneumococcus/resources.html. Accessed 11 August 2023.

[19] Neal EFG , Chan J, Nguyen CD, Russell FM. Factors associated with pneumococcal nasopharyngeal carriage: a systematic review. PLoS Glob Public Health 2022; 2:e0000327. 36962225 10.1371/journal.pgph.0000327 PMC10021834

[20] Hammitt LL , Akech DO, Morpeth SC, et al Population effect of 10-valent pneumococcal conjugate vaccine on nasopharyngeal carriage of *Streptococcus pneumoniae* and non-typeable *Haemophilus influenzae* in Kilifi, Kenya: findings from cross-sectional carriage studies. Lancet Glob Health 2014; 2:e397–405. 25103393 10.1016/S2214-109X(14)70224-4 PMC5628631

[21] Usuf E , Bottomley C, Adegbola RA, Hall A. Pneumococcal carriage in sub-Saharan Africa—a systematic review. PLoS One 2014; 9:e85001. 24465464 10.1371/journal.pone.0085001 PMC3896352

[22] Swarthout TD , Fronterre C, Lourenco J, et al High residual carriage of vaccine-serotype *Streptococcus pneumoniae* after introduction of pneumococcal conjugate vaccine in Malawi. Nat Commun 2020; 11:2222. 32376860 10.1038/s41467-020-15786-9 PMC7203201

[23] Spiegelman D , Hertzmark E. Easy SAS calculations for risk or prevalence ratios and differences. Am J Epidemiol 2005; 162:199–200. 15987728 10.1093/aje/kwi188

[24] Zou G . A modified Poisson regression approach to prospective studies with binary data. Am J Epidemiol 2004; 159:702–6. 15033648 10.1093/aje/kwh090

[25] Nzenze SA , von Gottberg A, Shiri T, et al Temporal changes in pneumococcal colonization in HIV-infected and HIV-uninfected mother-child pairs following transitioning from 7-valent to 13-valent pneumococcal conjugate vaccine, Soweto, South Africa. J Infect Dis 2015; 212:1082–92. 25784729 10.1093/infdis/jiv167

[26] Usuf E , Bottomley C, Gladstone R, et al Persistent and emerging pneumococcal carriage serotypes in a rural Gambian community after 10 years of pneumococcal conjugate vaccine pressure. Clin Infect Dis 2021; 73:e3825–35. 32584973 10.1093/cid/ciaa856

[27] Valenciano SJ , Moiane B, Lessa FC, et al Effect of 10-valent pneumococcal conjugate vaccine on *Streptococcus pneumoniae* nasopharyngeal carriage among children less than 5 years old: 3 years post-10-valent pneumococcal conjugate vaccine introduction in Mozambique. J Pediatric Infect Dis Soc 2021; 10:448–56. 33245124 10.1093/jpids/piaa132

[28] Childs L , Sawadogo G, Ouedraogo-Traore R, Medah I, Yameogo IIO, eds. Status of pneumococcal meningitis in Burkina Faso after 13-valent pneumococcal conjugate vaccine introduction and before a schedule change, 2018–2020 [abstract]. In: International Conference on Emerging Infectious Diseases, Atlanta, GA, 2022.

[29] Muller A , Kleynhans J, de Gouveia L, et al *Streptococcus pneumoniae* serotypes associated with death, South Africa, 2012–2018. Emerg Infect Dis 2022; 28:166–79. 34932448 10.3201/eid2801.210956 PMC8714227

[30] Kobayashi M , Bigogo G, Kim L, et al Impact of 10-valent pneumococcal conjugate vaccine introduction on pneumococcal carriage and antibiotic susceptibility patterns among children aged <5 years and adults with human immunodeficiency virus infection: Kenya, 2009–2013. Clin Infect Dis 2020; 70:814–26. 30959526 10.1093/cid/ciz285 PMC6942635

[31] Kwambana-Adams B , Hanson B, Worwui A, et al Rapid replacement by non-vaccine pneumococcal serotypes may mitigate the impact of the pneumococcal conjugate vaccine on nasopharyngeal bacterial ecology. Sci Rep 2017; 7:8127. 28811633 10.1038/s41598-017-08717-0 PMC5557800

[32] Nzenze SA , Shiri T, Nunes MC, et al Temporal changes in pneumococcal colonization in a rural African community with high HIV prevalence following routine infant pneumococcal immunization. Pediatr Infect Dis J 2013; 32:1270–8. 24061271 10.1097/01.inf.0000435805.25366.64

[33] van der Linden M , Perniciaro S, Imohl M. Increase of serotypes 15A and 23B in IPD in Germany in the PCV13 vaccination era. BMC Infect Dis 2015; 15:207. 25940580 10.1186/s12879-015-0941-9 PMC4424534

[34] Yildirim I , Lapidot R, Shaik-Dasthagirisaheb YB, et al Invasive pneumococcal disease after 2 decades of pneumococcal conjugate vaccine use. Pediatrics 2024; 153:e2023063039. 38087952 10.1542/peds.2023-063039

[35] Sheppard V . Research methods. New Westminster: Justice Institute of British Columbia; 2019. Available at: https://pressbooks.bccampus.ca/researchmethods/. Accessed 12 February 2024.

