# Supplementary material for: Pneumococcal Carriage in Burkina Faso After 13-Valent Pneumococcal Conjugate Vaccine Introduction and Before a Schedule Change
Source: Open Forum Infect Dis. 2024 May 31;11(6):ofae303. doi: 10.1093/ofid/ofae303 (PMC11191361; doi:10.1093/ofid/ofae303)
Supplement: ofae303_Supplementary_Data [file ofae303_supplementary_data.docx]

Supplemental Table 1 Pneumococcal Carriage Prevalence by Demographic and Epidemiological Characteristics of Enrolled Participants by Study Year, Bobo-Dioulasso, Burkina Faso, 2017 and 2020

| **Characteristics** | **2017** | | | | **2020** | | | | **Comparison in *S. pneumoniae* carriage prevalence in 2017 and 2020** |
| --- | --- | --- | --- | --- | --- | --- | --- | --- | --- |
|  | **Tested**  **N** | ***S. pneumoniae* detection**  **n (%)** | | ***P* Value^a^** | **Tested**  **N** | ***S. pneumoniae* detection**  **n (%)** | | ***P* Value^a^** | ***P* Value^b^** |
| **Sex** |  |  |  |  |  |  |  |  |  |
| Male | 446 | 281 | 63.0 | 0.163 | 454 | 279 | 61.5 | 0.203 | 0631 |
| Female | 559 | 328 | 58.7 |  | 548 | 315 | 57.5 |  | 0.687 |
| **Presence of other children <5 years of age in the household (other than the participant)^c^** |  |  |  | 0.007 |  |  |  | <0.0001 | 0.680 |
| Yes | 943 | 582 | 61.7 |  | 889 | 557 | 62.7 |  |  |
| No | 61 | 27 | 44.3 |  | 113 | 37 | 32.7 |  |  |
| **Household size^c^** |  |  |  |  |  |  |  |  |  |
| 1-3 | 149 | 79 | 53.0 | 0.065 | 141 | 78 | 55.3 | 0.357 | 0.695 |
| 4-6 | 423 | 270 | 63.8 |  | 519 | 304 | 58.6 |  | 0.100 |
| >6 | 432 | 260 | 60.2 |  | 342 | 212 | 62.0 |  | 0.610 |
| **≥4 persons sharing a room^d^** |  |  |  | 0.0004 |  |  |  | 0.001 | 0.801 |
| Yes | 151 | 111 | 73.5 |  | 133 | 96 | 72.2 |  |  |
| No | 851 | 495 | 58.2 |  | 869 | 498 | 57.3 |  |  |
| **Child in household attending daycare or school^d^** |  |  |  | 0.787 |  |  |  | 0.602 | 0.660 |
| Yes | 763 | 464 | 60.8 |  | 777 | 464 | 59.7 |  |  |
| No | 239 | 143 | 59.8 |  | 225 | 130 | 57.8 |  |  |
| **Cigarette smoker in household^d^** |  |  |  | 0.999 |  |  |  | 0.873 | 0.892 |
| Yes | 208 | 126 | 60.6 |  | 152 | 91 | 59.9 |  |  |
| No | 794 | 481 | 60.6 |  | 850 | 503 | 59.2 |  |  |
| **Fuel source – gas^e^** |  |  |  |  |  |  |  |  |  |
| Yes | 312 | 167 | 53.5 | 0.002 | 115 | 56 | 48.7 | 0.014 | - |
| No | 693 | 442 | 63.8 |  | 887 | 538 | 60.7 |  |  |
| **Fuel source – coal^e^** |  |  |  | 0.001 |  |  |  | 0.602 | - |
| Yes | 835 | 525 | 62.9 |  | 709 | 424 | 59.8 |  |  |
| No | 170 | 84 | 49.4 |  | 293 | 170 | 58.0 |  |  |
| **Fuel source – wood^e^** |  |  |  | 0.417 |  |  |  | 0.154 | - |
| Yes | 488 | 302 | 61.9 |  | 178 | 114 | 64.0 |  |  |
| No | 517 | 307 | 59.4 |  | 824 | 480 | 58.3 |  |  |
| **Cooking location – inside** |  |  |  | 0.554 |  |  |  | 0.042 | 0.356 |
| Yes | 304 | 180 | 59.2 |  | 455 | 254 | 55.8 |  |  |
| No | 701 | 429 | 61.2 |  | 547 | 340 | 62.2 |  |  |
| **Cooking location – under hangar** |  |  |  | 0.312 |  |  |  | 0.769 | 0.810 |
| Yes | 141 | 80 | 56.7 |  | 141 | 82 | 58.2 |  |  |
| No | 864 | 529 | 61.2 |  | 861 | 512 | 59.5 |  |  |
| **Cooking location – outside** |  |  |  | 0.215 |  |  |  | 0.066 | 0.889 |
| Yes | 757 | 467 | 61.7 |  | 662 | 406 | 61.3 |  |  |
| No | 248 | 142 | 57.3 |  | 340 | 188 | 55.3 |  |  |
| **Household possessions – radio** |  |  |  | 0.195 |  |  |  | 0.770 | 0.280 |
| Yes | 753 | 465 | 61.8 |  | 717 | 423 | 59.0 |  |  |
| No | 252 | 144 | 57.1 |  | 285 | 171 | 60.0 |  |  |
| **Household possessions – television** |  |  |  | 0.739 |  |  |  | 0.743 | 0.593 |
| Yes | 830 | 501 | 60.4 |  | 885 | 523 | 59.1 |  |  |
| No | 175 | 108 | 61.7 |  | 117 | 71 | 60.7 |  |  |
| **Household possessions – phone** |  |  |  | 0.768 |  |  |  | 0.033 | 0.653 |
| Yes | 983 | 595 | 60.5 |  | 996 | 593 | 59.5 |  |  |
| No | 22 | 14 | 63.6 |  | 6 | 1 | 16.7 |  |  |
| **Household possessions – motorbike** |  |  |  | 0.531 |  |  |  | 0.766 | 0.669 |
| Yes | 810 | 487 | 60.1 |  | 863 | 510 | 59.1 |  |  |
| No | 195 | 122 | 62.6 |  | 139 | 84 | 60.4 |  |  |
| **Illness in the past two weeks – cold/runny nose** |  |  |  | 0.0009 |  |  |  | <0.0001 | 0.735 |
| Yes | 474 | 313 | 66.0 |  | 531 | 356 | 67.0 |  |  |
| No | 531 | 296 | 55.7 |  | 471 | 238 | 50.5 |  |  |
| **Illness in the past two weeks – cough** |  |  |  | 0.043 |  |  |  | 0.002 | 0.581 |
| Yes | 376 | 243 | 64.6 |  | 297 | 198 | 66.7 |  |  |
| No | 629 | 366 | 58.2 |  | 705 | 396 | 56.2 |  |  |
| **Illness in the past two weeks – fever** |  |  |  | 0.268 |  |  |  | 0.553 | 0.647 |
| Yes | 208 | 133 | 63.9 |  | 143 | 88 | 61.5 |  |  |
| No | 797 | 476 | 59.7 |  | 859 | 506 | 58.9 |  |  |
| **Antibiotic use in the past two weeks** |  |  |  | 0.425 |  |  |  | 0.102 | 0.762 |
| Yes | 114 | 73 | 64.0 |  | 129 | 85 | 65.9 |  |  |
| No | 891 | 536 | 60.2 |  | 873 | 509 | 58.3 |  |  |

^a^χ^2^ tests used to compare proportions of pneumococcal carriers by demographic and epidemiological characteristics within the same study year.

^b^χ^2^ tests were used to compare proportions of pneumococcal carriers by demographic and epidemiological characteristics in 2017 vs. 2020.

^c^One response missing from 2017 study (N=1004).

^d^Three responses missing from 2017 study (N=1002).

^e^Multiple responses possible in 2017 study while one response only possible in 2020 study. *P* value not calculated due to differences in the question format.

Supplemental Table 2. Pneumococcal Carriage Prevalence of PCV13 Vaccine Serotypes by Age Group, Bobo-Dioulasso, Burkina Faso, 2017 and 2020

| **PCV13 vaccine serotypes** | **Age group^a^** | | | | | | | | | |
| --- | --- | --- | --- | --- | --- | --- | --- | --- | --- | --- |
|  | **<1 year** | | **1 year** | | **2-4 years** | | **5-14 years** | | **≥15 years** | |
|  | **2017**  **N=201** | **2020**  **N=200** | **2017**  **N=199** | **2020**  **N=200** | **2017**  **N=204** | **2020**  **N=201** | **2017**  **N=201** | **2020**  **N=202** | **2017**  **N=200** | **2020**  **N=199** |
|  | **n (%)** | **n (%)** | **n (%)** | **n (%)** | **n (%)** | **n (%)** | **n (%)** | **n (%)** | **n (%)** | **n (%)** |
| 1 | 1 (0.5) | 0 (0.0) | 0 (0.0) | 0 (0.0) | 0 (0.0) | 0 (0.0) | 0 (0.0) | 0 (0.0) | 1 (0.5) | 0 (0.0) |
| 3 | 4 (2.0) | 3 (1.5) | 8 (4.0) | 3 (1.5) | 8 (3.9) | 4 (2.0) | 17 (8.5) | 13 (6.4) | 3 (1.5) | 3 (1.5) |
| 4 | 1 (0.5) | 1 (0.5) | 0 (0.0) | 2 (1.0) | 1 (0.5) | 2 (1.0) | 3 (1.5) | 1 (0.5) | 3 (1.5) | 1 (0.5) |
| 5 | 0 (0.0) | 1 (0.5) | 0 (0.0) | 0 (0.0) | 0 (0.0) | 0 (0.0) | 0 (0.0) | 2 (1.0) | 1 (0.5) | 0 (0.0) |
| 6A | 5 (2.5) | 4 (2.0) | 4 (2.0) | 4 (2.0) | 4 (2.0) | 1 (0.5) | 6 (3.0) | 6 (3.0) | 1 (0.5) | 1 (0.5) |
| 6B | 2 (1.0) | 2 (1.0) | 4 (2.0) | 4 (2.0) | 3 (1.5) | 6 (3.0) | 5 (2.5) | 1 (0.5) | 1 (0.5) | 2 (1.0) |
| 7F | 0 (0.0) | 1 (0.5) | 0 (0.0) | 2 (1.0) | 0 (0.0) | 2 (1.0) | 0 (0.0) | 1 (0.5) | 0 (0.0) | 0 (0.0) |
| 9V | 2 (1.0) | 1 (0.5) | 0 (0.0) | 0 (0.0) | 1 (0.5) | 2 (1.0) | 2 (1.0) | 1 (0.5) | 2 (1.0) | 0 (0.0) |
| 14 | 2 (1.0) | 4 (2.0) | 2 (2.0) | 6 (3.0) | 3 (1.5) | 3 (1.5) | 4 (2.0) | 1 (0.5) | 2 (1.0) | 1 (0.5) |
| 18C | 0 (0.0) | 1 (0.5) | 5 (2.5)^b^ | 0 (0.0)^b^ | 2 (1.0) | 2 (1.0) | 2 (1.0) | 3 (1.5) | 1 (0.5) | 1 (0.5) |
| 19A | 4 (2.0) | 4 (2.0) | 2 (1.0) | 6 (3.0) | 0 (0.0)^b^ | 6 (3.0)^b^ | 5 (2.5) | 3 (1.5) | 2 (1.0) | 1 (0.5) |
| 19F | 15 (7.5) | 12 (6.0) | 7 (3.5) | 10 (5.0) | 13 (6.4) | 10 (5.0) | 9 (4.5)^b^ | 2 (1.0)^b^ | 7 (3.5)^b^ | 0 (0.0)^b^ |
| 23F | 12 (6.0)^b^ | 4 (2.0)^b^ | 9 (4.5)^b^ | 2 (1.0)^b^ | 11 (5.4)^b^ | 0 (0.0)^b^ | 7 (3.5)^b^ | 0 (0.0)^b^ | 0 (0.0) | 1 (0.5) |
| Total | 48 (23.9) | 38 (19.0) | 41 (20.6) | 39 (19.5) | 46 (22.6) | 38 (18.9) | 58 (28.9)^b,c^ | 33 (16.3)^b,c^ | 24 (12.0)^b^ | 11 (5.5)^b^ |

PCV13: 13-valent pneumococcal conjugate vaccine

^a^χ^2^ tests and Fisher’s exact tests used to compare changes in individual vaccine serotype carriage prevalence in each age group between 2017 and 2020.

^b^*P* value <0.05

^c^Two participants in 2017 and one participant in 2020 were colonized with two vaccine serotypes, therefore, the number of participants colonized with individual vaccine serotypes in each column may exceed the total participants colonized with any vaccine serotype.

Supplemental Table 3. Availability of PCV13 vaccination history among children <5 years of age in 2017 and <7 years of age in 2020, Bobo-Dioulasso, Burkina Faso, 2017 and 2020

| **PCV13 vaccination status** | **2017^a^**  **N=602^b^** | | **2020**  **N=664^b,c^** | |
| --- | --- | --- | --- | --- |
|  | **n** | **%** | **n** | **%** |
| Confirmed by vaccination card or registry | 387 | 64.3 | 536 | 80.7 |
| Confirmed by caregiver verbal report only | 18 | 3.0 | 52 | 7.8 |
| Not vaccinated | 105 | 17.4 | 64 | 9.6 |
| Don’t know | 92 | 15.3 | 12 | 1.8 |

PCV13: 13-valent pneumococcal conjugate vaccine

^a^Since PCV13 was introduced in 2013, children ≥5 years were not eligible for PCV13 vaccination in 2017.

^b^Responses missing from two participants in 2017 and 2020.

^c^Children aged 5 and 6 years are included in the 5–14-year-old age group. There were 65 children 5 or 6 years old enrolled in 2020.
